# Serum adipocytokines and adiposity as predictive indices of preeclampsia

**DOI:** 10.1186/s40885-020-00152-0

**Published:** 2020-10-01

**Authors:** Ahmed Tijani Bawah, Francis Agyemang Yeboah, Salifu Nanga, Huseini Alidu, Robert A. Ngala

**Affiliations:** 1 Department of Medical Laboratory Science, School of Allied Health Sciences, University of Health and Allied Health Sciences, PMB 31, Ho, Ghana; 2 Department of Molecular Medicine, Kwame Nkrumah University Science and Technology, Kumasi, Ghana; 3 School of Basic and Biomedical Science, University of Health and Allied Sciences, Ho, Ghana

**Keywords:** Preeclampsia, Adiponectin, Leptin, Resistin, Visfatin

## Abstract

**Background:**

This study was aimed at determining the levels of serum adiponectin, leptin, resistin, visfatin and lipids during the first trimester in pregnant women and to evaluate the relationship between these biochemical markers and preeclampsia (PE). Available evidence point to changes in the levels of these adipokines in PE hence this study examined the potential of using these biomarkers in the prediction of the disease.

**Methods:**

This was a case-control study which compared first trimester serum biochemical and anthropometric parameters in pregnant women who subsequently developed PE and those who did not. Blood pressure and urine protein were determined after 20 weeks of gestation and diagnosis of PE performed according to the guidelines of the American Heart Association.

**Results:**

There was no significant difference (*p* > 0.05) in the lipid profile with the exception of HDL cholesterol which was significantly lower (*p* = 0.043) in the PE group compared to the normotensive group. There were, however, significant differences (*p* <  0.05) in the adipokines between the PE group and those without PE. Analyses of area under the receiver operating characteristic curves (AUCs) for the adipokines, showed their ability to correctly predict PE even after controlling for body mass index (BMI) and family history of hypertension.

**Conclusion:**

Adiponectin, leptin, resistin and visfatin were found to be significant predictors of PE, with resistin being the best predictor after controlling for BMI. However, adiponectin was the best predictor after controlling for BMI, age, parity and family history of diabetes and preeclmapsia.

## Background

The main objective of the current study was to explore the association between some adipokines, lipid and preeclampsia and to elucidate the effectiveness and accuracy of these markers in the prediction of PE. PE, a pregnancy-specific syndrome [1] is the occurrence of hypertension and proteinuria after 20 weeks of gestation in women who were previously normotensive [2]. It affects 2–5% of pregnancies and is a major contributor to fetal, neonatal and maternal morbidity and mortality. The incidence rate in Ghana is about 7% [3, 4], however a prevalence of 8.3% was reported in a study at the Volt Regional Hospital, Ho [5]. Preeclampsia may develop from 20 weeks of gestation up to 6 weeks postpartum and is usually regarded as an early inception if it occurs before 34 weeks of gestation. Preeclampsia shares some of the risk factors of metabolic syndrome including insulin resistance, subclinical inflammation and obesity with evidence showing that women with PE have an increased risk of developing cardiovascular disease in the future [6].

Some studies have reported a paradoxical and significant increase in adiponectin concentration during pregnancy complicated by PE [7, 8]. However, other authors did not find significant difference in adiponectin mRNA expression in adipose tissue between patients with PE and healthy controls [9].

Leptin levels are increased in pregnant women with PE [7] and may be increased before the clinical onset of the disease [10, 11] with peaks occurring around 28 weeks of gestation [12]. Therefore, leptin may be involved in the pathophysiology of the disease. Some authors have, however, reported decreased [9] or unchanged [13] circulating levels in patients with PE.

Resistin concentration in PE has been reported by various researchers to remain unchanged [7] decreased [14] or increased [9]. The elevated circulating resistin levels in PE may be due to the fact that its concentration in plasma depends on glomerular filtration hence with progressive renal impairment in PE resistin levels in plasma might increase [15].

Similar conflicting results have been published on visfatin levels during pregnancy complicated by preeclampsia. Some authors published increased visfatin levels in PE [16] while other investigators reported decreased levels [17] or values similar to normal pregnancy [18]. This study was aimed at determining whether in the first trimester of pregnancy, serum leptin, adiponectin, resistin and visfatin are altered in pregnancies that subsequently develop PE and whether these adipokines and lipids can be used as predictive tools to ascertain pregnancies that are likely to develop PE.

Lipid profile changes in normal pregnancy are regarded as discernable elevations of total plasma cholesterol and triglyceride levels as a result of increased synthesis of triglycerides (TG) by the liver and very low density lipoprotein-cholesterol (VLDL-C) in response to elevated estrogen levels [19]. Reduction in Lipoprotein lipase (LPL) activity due to the down regulation of LPL gene expression by estrogen during pregnancy reduces the clearance of VLDL-C [20]. A study conducted in the Cape Coast metropolis in Ghana revealed that women with PE had elevated TG, total cholesterol (TC), low density lipoprotein cholesterol (LDL-C) and VLDL-C [21]. The variation in the lipid profile and changes in some adipokine metabolism as reported by various investigators calls for a close scrutiny of their roles in the pathogenesis of PE. The main aim of this study was to determine whether in the first trimester of pregnancy, metabolism of adiponectin, leptin resistin and visfatin are affected in pregnancies that subsequently develop PE and whether these changes are significant enough in the prediction of PE so as to elicit interventions early enough to save the mother and the baby.

## Materials and methods

### Study site and design

This case-control study was carried out at the Ho Teaching Hospital (HTH) Ho, Ghana, between January, 2016 and December, 2016.

### Criteria for selection

Those included in the study were pregnant women above 18 years with or without hypertension (cases and controls respectively). Pregnant women without dipstick proteinuria and whose blood pressure < 140/90 mmHg were enrolled as controls while those with concurrent hypertension and proteinuria were enrolled as cases. Those excluded were pregnant women with renal disease, diabetes, cancers multiple pregnancy and pre-gestational hypertension.

### Study population

We compared first trimester parameters among 90 pregnant women who subsequently developed PE and 100 women who did not developed the condition at VRH. Participants were selected from a large prospective observational study for early prediction of pregnancies that are likely to develop complications in women attending antenatal clinic at VRH. Women, who visited the hospital between 11 and 13 weeks of gestation, were recruited to take part in the study. We recorded maternal characteristics and medical history of participants.

### Anthropometric measurement

The participants wore light clothing and stood on a Bio impedance analyzer (BIA) (BIA; BSD01, Pure Pleasure, a division of the Stingray Group, Cape town, South Africa) after they had removed their shoes; their weights were recorded to the nearest 0.1 kg. Height was measured without shoes with a stadiometer to the nearest 0.5 cm with the study participants standing upright and heels put together and the head in the horizontal plane. BMI was calculated as weight/height squared (Kg/m^2^).

### Blood pressure measurement

Each participant was asked to sit down comfortably, extend the left arm on a table and then relax for 10 min. Blood pressure was measured using a mercury sphygmomanometer and stethoscope. Measurements were taken from the left upper arm after the participants had rested for at least 5 min in accordance with the guidelines of the American Heart Association [22]. Triplicate measurements were taken with at least 5 min waiting period between measurements and the mean blood pressure was recorded to the nearest 2.0 mmHg.

### Collection and preservation of samples

During the first trimester, 5 milliliter of blood samples were also taken between 7:00 am and 8:00 am and put into serum separator tubes and immediately put on ice packs. Serum samples were separated within 1 h and then stored in several aliquots at -80 °C for subsequent biochemical analysis. After 20th week of gestation, each participant was provided with clean dry, wide mouth, leak proof containers to collect about 5 ml of urine sample.

### Biochemical and urine analysis

Adiponectin, leptin, resistin and visfatin were analyzed in the base line samples of both cases and the controls by sandwich enzyme-linked immunosorbent assay technique (Elabscience Biotechnology Co. Ltd., Wu Han, People’s Republic of China) whiles lipid profile was done using the Vitros dry chemistry analyzer (Ortho-Clinical Diagnostics, Johnson & Johnson, High Wycombe, UK). None of the samples in this study were previously thawed and refrozen.

Urine strip was inserted into the urine sample up to the test area for less than 2 s. The edges of the strips were drawn along the brims of the vessels to remove excess urine, making sure the tests areas did not touch the vessels. The strips were held vertical and the tips tapped on absorbent papers to remove any remaining urine [23]. The urine strip was held horizontally and compared with the color chart on the vial label under bright light. The amount of protein was then determined using the intensity of the blue green color which was proportional to concentration of protein in the urine. Proteinuria was defined as the presence of urinary protein with concentration of at least “+” [24].

### Study variables and outcome measurement

Every pregnant woman in this hospital is screened for PE after the 20th week of gestation. The primary outcome was PE occurrence (yes/no), which was determined according to the PE diagnosis criteria. Blood pressure measurement was done after 20 weeks of pregnancy and urine protein determined during the same period using the dip-stick qualitative/semi-quantitative method (Urit Medical Electronic Co., Ltd., Guangxi, People’s Republic of China). Diagnosis of PE was performed by qualified Obstetrician/Gynecologist based on systolic and diastolic blood pressure of 140 mmHg or more and 90 mmHg or more respectively (or both) on two occasions at least 4 h apart accompanied by proteinuria of + or more.

### Statistical analysis

Data analysis was performed with the SPSS software, version 20. Systat, Inc. Germany and Graph Pad Prism, version 5.0, San Diego California, USA. Normality test for variables under study was performed by Shapiro-Wilk test followed by comparison between those with PE and the normotensives by Mann–Whitney *U*-test. In all the statistical analysis, a value of *p* <  0.05 was considered to be significant. The area under the receiver operating characteristic (ROC) curve (AUC) is mostly used to measure the accuracy of a test/marker. When the AUC is 50% or less, the result can be regarded as a random guessing and hence not significant. This is represented by diagonal line in the ROC plot [25] . We tested the adipokines and the lipids for their accuracy (AUC ~ 60%) in determining pregnancies that are likely to develop into preeclampsia.

Multivariate analysis was carried out for the individual adipokines as predictors of PE after controlling for potential confounding variables (Age, BMI, relatives with hypertension, family history of diabetes mellitus, family history of preeclampsia and parity). This was to assess the independent contribution of each adipokine in predicting PE after controlling for the confounders.

The statistics -2Log (Likelihood), R^2^ (Cox and Snell), R^2^ (Nagelkerke), Akaike Information Criterion (AIC) and Correct Classification Rate (CCR) were considered as the parameters for goodness of fit test for the models. The -2Log (Likelihood) statistic measures how poorly a model predicts an event of interest, the smaller the statistic the better the model. The Cox and Snell R^2^ and Nagelkerke R^2^ are coefficient of determination used to estimate the proportion of variance in the dependent variable which is explained by the independent variable. The Nagelkerke R^2^ is an adjusted version of the Cox and Snell R^2^. The AIC is also an estimator of the relative quality of statistical models. The smaller the estimate, the better the model. Another useful measure to assess the utility of a logistic regression model is the Correct Classification Rate (CCR).

## Results

The baseline demographics, lipids and adipokine characteristics were compared among the outcome group (PE and No PE) (Table 1). The mean age of those with PE was significantly higher than those without PE (35.1 vs 28.44 years; *P <* 0.0001); BMI was also significantly higher in those who developed PE than in those who did not (32.63 vs 24.99 kg/m^2^; *P* <  0.0001). The lipid profile parameters did not show any significant difference between the PE group and those without PE, except HDL which was significantly lower in the PE group compared to those without PE (1.39 vs 1.569, *p* = 0.043) (Table 1). Leptin (39.26 vs 18.46 ng/mL, *P* <  0.0001) was statistically significantly higher in the PE compared those without PE. Similarly, resistin and visfatin were significantly higher in the PE group compared to the normotensives (*p* <  0.0001) while adiponectin was significantly lower in the PEs compared to the non PEs (Table 1).

**Table 1:** Mann-Whitney U test for base line biochemical markers and maternal characteristics for study participants

Var.	BMI		Age		ADP		LP		RTN		VF		TG		TC		HDL		LDL		VLDL	
PE	No	Yes	No	Yes	No	Yes	No	Yes	No	Yes	No	Yes	No	Yes	No	Yes	No	Yes	No	Yes	No	Yes
Min.	18.9	27.1	16	24	17.7	15.2	2.7	9.9	1.3	4.0	0.3	0.5	0.5	0.5	2.4	2.6	0.1	0.1	0.2	0.2	0.2	0.2
Max.	37.3	37.3	41	46	258.2	90.6	40.7	41	12.4	13.9	11.2	19.4	3.3	3.4	9.6	9.3	3.4	3.4	7.8	7.8	1.5	1.4
Mean	25.0	32.6	28.4	35.1	83.6	39.3	18.5	36.3	6.4	10.2	4.4	7.4	1.7	1.6	5.8	5.9	1.6	1.4	3.8	4.0	0.8	0.7
LB	24.2	32.0	27.4	34.0	77.3	35.3	16.8	34.7	6.0	9.8	3.9	6.7	1.5	1.5	5.5	5.5	1.4	1.2	3.5	3.6	0.7	0.6
UB	25.8	33.2	29.5	36.2	90.0	43.2	20.2	37.9	6.8	10.7	5.0	8.0	1.8	1.8	6.1	6.3	1.7	1.7	4.1	4.4	0.9	0.8
*P* Value	< 0.0001**		< 0.0001**		< 0.0001**		< 0.0001**		< 0.0001**		< 0.0001**		0.86		0.826		0.043*		0.589		0.73	

**significant at 0.01 level

*significant at 0.05 level

*ADP* Adiponectin, *LP* Leptin, *RTN* Resistin, *VF* Visfatin, *TG* Triglyceride, *TC* Total Cholesterol, *LDL* Low Density Lipoprotein Cholesterol, *HDL* High Density Lipoprotein Cholesterol, *LDL* Low Density Lipoprotein, *VLDL* Very Low Density Lipoprotein Cholesterol

Performance of the screening results was examined using the ROC curves. The areas under the ROC curve and the sensitivities and specificities as well as threshold points for the detection of PE are given in Table 2. In this study, the accuracy with which the biochemical markers are able to discriminate on the status of PE was evaluated. Thus, in Table 2, the following adipokines with the corresponding AUCs; Leptin (92.0%), Resistin (91.4%), and Adiponectin (90.5%) have excellent accuracy level while Visfastin (77.1%) has fair accuracy level respectively of determining the presence of PE per Table 2 ratings. Adiponectin demonstrated sensitivity and specificity of 87.8 and 86% respectively with a cut-off point ≤50.55 ng/ml while leptin showed both sensitivity and specificity of 92%, with threshold of ≥27 ng/ml. Furthermore, Resistin showed sensitivity and specificity of 94 and 91% respectively and a cut-off point about 9 ng/ml, while visfatin had sensitivity and specificity of 69 and 83% respectively with a threshold of ≥6.67 ng/ml. This indicates that adiponectin, leptin, resistin and visfatin are good predictors of PE (Table 2).

**Table 2:** AUC, Sensitivity, Specificity and threshold point for the Adipokines in the pregnant women

Variables	AUC (%)	Sensitivity (%)	Specificity (%)	Threshold point
ADP	90.5	87.8	86	≤50.552
LP	92	92.2	92	≥ 27.273
RTN	91.4	94.4	91.4	≥ 8.949
VF	77.1	68.9	83	≥ 6.667

*ADP* Adiponectin, *LP* Leptin, *RTN* Resistin and *VF* Visfatin

**Fig. 1:**
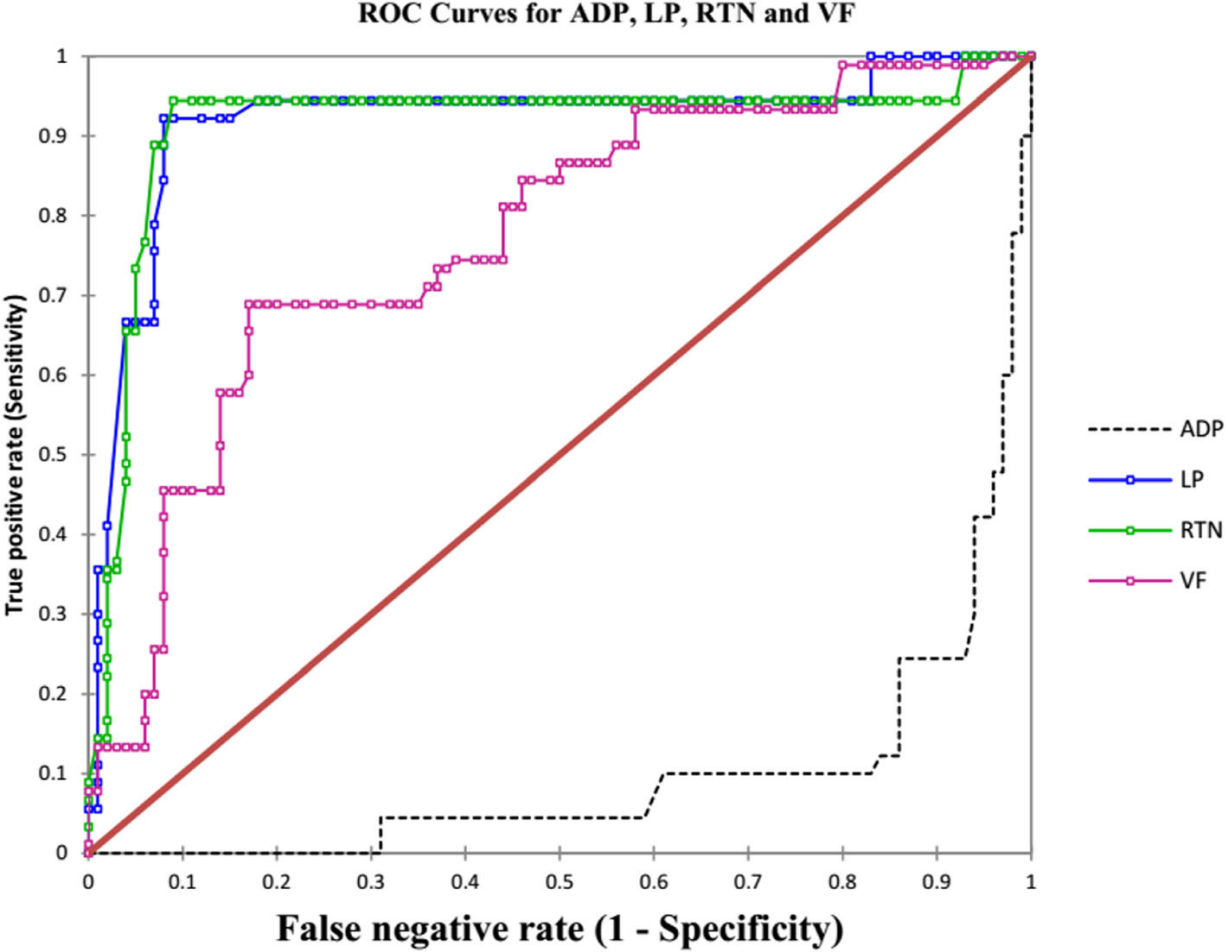
ROC curves for the adipokines. AUCs (%): ADP (95.0), LP (92.0) RTN (91.4) and VF (77.1) ADP = Adiponectin, LP = Leptin, RTN = Resistin, VF = Visfatin

Additionally, a close look at the ROC plots (Fig. 1) reveals that all of them are far from the diagonal line which represents 50% and therefore not random guessing but significant. This suggests that they have high accuracy in determining pregnancies that are likely to develop PE. After controlling for BMI, none of those within the normal BMI category developed PE (Table 3); consequently, no values for AUCs were recorded for all the adipokines analysed. However, the overweight group showed higher AUCs, sensitivities and specificities. The obese group however showed reduced sensitivities and specificities. These results points to possible influence of body mass index on adiponectin leptin resistin and visfatin but it also points to possible negative feedback mechanism in the metabolism of these adipocytokine during pregnancy, but more importantly BMI does not appear have effect on the predictive ability of these signalling molecules of PE.

**Table 3:** AUC, Sensitivity, Specificity and threshold point for levels of the Adipokines in the pregnant women controlling for BMI

Adipokine	BMI Category	Prevalence (%)	AUC (%)	Sensitivity (%)	Specificity (%)	Threshold point
ADP	Normal Weight	0	–	–	–	–
	Overweight	23	97.7	100	97.7	≤ 36.163
	Obese	89	83.2	71.4	90	≤ 44.980
LP	Normal Weight	0	–	–	–	–
	Overweight	23	99.3	100	97.7	≥ 27.245
	Obese	89	70.7	61	80	≥ 38.482
RTN	Normal Weight	0	–	–	–	–
	Overweight	23	95.5	100	90.9	≥ 8.949
	Obese	89	93.5	93.5	70	≥ 8.949
VF	Normal Weight	0	–	–	–	–
	Overweight	23	89.5	84.6	90.9	≥ 6.628
	Obese	89	48.2	66.2	60	≥ 6.243

*ADP* Adiponectin, *LP* Leptin, *RTN* Resistin and *VF* Visfatin

BMI classification: Normal Weight = (18.5–24.9 kg/m^2^), Overweight = (25.0–29.99 kg/m^2^), Obese = (Above 30.0 kg/m^2^)

After controlling for family history of hypertension which is a known confounding factor, there were variations in the AUCs, sensitivities and specificities as well as the threshold points for predicting PE but these variations were minimal and the overall effect of the predictive abilities of these adipocytokines remained intact (Table 4).

**Table 4:** AUC, Sensitivity, Specificity and threshold point for the Adipokines in the pregnant women controlling for those who have relatives with hypertension

		Prevalence (%)	AUC (%)	Sensitivity (%)	Specificity (%)	Threshold point
	RWHP					
ADP	YES	73	83.6	84.2	78.6	≤ 50.552
	NO	38	92.2	84.6	95.3	≤ 44.980
LP	YES	73	90.6	89.5	92.9	≥ 25.611
	NO	38	92.2	94.2	91.9	≥ 27.273
RTN	YES	73	89.8	89.5	85.7	≥ 9.012
	NO	38	91.6	94.2	93	≥ 8.949
VF	YES	73	72.5	78.9	78.6	≥ 6.349
	NO	38	75.1	61.5	83.7	≥ 6.667

*ADP* Adiponectin, *LP* Leptin, *RTN* Resistin and *VF* Visfatin, *RWHP* Relatives with hypertension

YES = those who have relatives with hypertension, NO = those who do not have relatives with hypertension

Multivariate analysis of individual adipokines as predictors of PE is shown in Table 5. Models 1, 2, 3 and 4 included adiponectin, leptin, resistin and visfatin respectively as predictors whiles controlling for confounding factors like age, parity, BMI, relative with hypertension, and family history of diabetes and preeclampsia. Model 1 with adiponectin as a predictor was the best model based on the criteria considered. This includes having the highest Nagelkerke R^2^ and CCR statistics of 95 and 95.26%, implying that adiponectin is the best predictor of PE after controlling for the confounders. Model 4 with visfatin as predictor had the least predictive ability with Nagelkerke R^2^ and CCR statistics of 89 and 91.58% respectively. The predictive ability of all the models was generally good (Table 5).

**Table 5:** Multivariate analysis of clinical factors affecting preeclampsia

	**Model 1(ADP)**			**Model 2(LP)**			**Model 3(RTN)**			**Model 4(VF)**		
	***P*-Value**	**OR**	**OR CI(95%)**	***P*- Value**	**OR**	**OR CI (95%)**	***P*-Value**	**OR**	**OR CI (95%)**	***P*-Value**	**OR**	**OR CI (95%)**
Intercept	0.50			**< 0.0001**			**< 0.0001**			< 0.0001		
ADP	**< 0.0001**	0.93	(0.9, 0.96)									
LP				**< 0.0001**	1.15	(1.08, 1.22)						
RTN							**< 0.0001**	1.65	(1.3, 2.11)			
VF										0.01	1.28	(1.07, 1.55)
**Age Cat.**												
20 To 35 years												
Above 35 years	**0.04**	8.86	(1.16, 67.68)	0.07	4.60	(0.88, 24.08)	**0.01**	6.25	(1.46, 26.69)	**0.01**	5.67	(1.41, 22.74)
Less than 20 years	0.89	1.39	(0.01, 135.01)	0.12	32.85	(0.41, 2604.47)	0.39	7.86	(0.07, 901.18)	0.12	27.92	(0.41, 1895.49)
**BMI Category**												
Overweight												
Obese	**< 0.0001**	44.24	(7.5, 260.91)	**< 0.0001**	20.43	(4.75, 87.87)	**< 0.0001**	19.88	(5.06, 78.17)	**< 0.0001**	26.52	(7.73, 90.98)
Normal Weight	0.16	0.12	(0.01, 2.29)	0.20	0.15	(0.01, 2.62)	0.10	0.08	(0, 1.67)	0.06	0.07	(0, 1.07)
**Parity**												
1												
2	0.84	1.18	(0.22, 6.41)	0.19	2.76	(0.6, 12.76)	0.08	4.00	(0.84, 19.14)	0.16	2.75	(0.67, 11.2)
3	**0.02**	11.34	(1.53, 84.29)	0.02	7.51	(1.29, 43.58)	0.13	3.73	(0.69, 20.11)	0.05	4.40	(0.99, 19.5)
> 4	**0.04**	9.07	(1.03, 80.17)	**< 0.0001**	17.35	(2.72, 110.49)	**< 0.0001**	14.38	(2.45, 84.57)	**< 0.0001**	12.19	(2.41, 61.59)
**Family Hist. of Hyp.**												
No												
Yes	0.19	2.93	(0.59, 14.48)	**0.04**	4.58	(0.97, 21.64)	0.10	3.84	(0.79, 18.77)	0.07	3.43	(0.89, 13.21)
**Family Hist. of DM**												
No												
Yes	0.40	2.23	(0.35, 14.34)	0.76	1.28	(0.26, 6.21)	0.72	0.75	(0.16, 3.53)	0.88	0.90	(0.21, 3.77)
**Family Hist. of PE**												
No												
Yes	0.79	0.82	(0.19, 3.52)	0.11	0.34	(0.09, 1.26)	0.22	0.45	(0.12, 1.62)	0.14	0.41	(0.12, 1.36)
**Goodness of Fit Statistics**												
	**Model 1**			**Model 2**			**Model 3**			**Model 4**		
-2Log(Likelihood)	27.186			37.24			40.66			54.11		
R^2^(Cox and Snell)	0.71			0.70			0.69			0.67		
R^2^(Nagelkerke)	0.95			0.93			0.92			0.89		
AIC	51.19			61.24			64.66			78.11		
CCR(%)	95.26			93.16			92.63			91.58		

Adiponectin, leptin, resistin and visfatin were found to be significant predictors of PE in their respective models (*P* <  0.05). Taking the reciprocal of the odds ratio, the odds of PE increases by a factor of 1.1 for each unit decrease in adiponectin (Model 1). A unit increase in leptin will increase the odds of PE by a factor of 1.15 (Model 2). Also, a unit increase in resistin will increase the odds of PE by a factor of 1.65 (Model 3) while visfatin increases the odds of PE by a factor of 1.28 (Model 4).

Obesity with overweight as reference category under BMI and Parity of four and above with parity one as reference category were found to be a significant confounders in all the four models. Age category above 35 years with age group 20 to 35 years as reference category was also found to be significant in models 1, 3 and 4.

Adiponectin showed positive correlation with HDL and negative correlation with TG and VLDL but these correlations were very weak. Leptin and resistin also showed very weak negative correlations with HDL, however, visfatin had significant negative correlation with HDL. Adiponectin correlated negatively with leptin, resistin, and visfatin. However, there were positive correlations between leptin, resistin and visfatin (Table 6). These correlations were very weak in those with normal weight but more pronounced in the overweight and obese categories (Table 7). This reemphasizes the link between adiposity and some of these adipokines.

**Table 6:** Correlation among Adipokines and Lipids

Variables	ADP	LP	RTN	VF	TC	HDL	LDL	VLDL	TG
ADP	**1**	**−0.5403**	**−0.3807**	**−0.2399**	0.0549	0.0531	0.0390	−0.0640	−0.0558
LP	**−0.5403**	**1**	**0.6667**	**0.5460**	0.0629	−0.1333	0.0963	−0.0016	0.0092
RTN	**−0.3807**	**0.6667**	**1**	**0.4510**	0.0911	− 0.1248	0.0671	0.0227	0.0395
VF	**− 0.2399**	**0.5460**	**0.4510**	**1**	**0.1928**	**−0.1527**	**0.1699**	0.1252	**0.1437**
TC	0.0549	0.0629	0.0911	**0.1928**	**1**	**−0.3359**	**0.8891**	**0.5973**	**0.5912**
HDL	0.0531	−0.1333	−0.1248	**− 0.1527**	**−0.3359**	**1**	**−0.3899**	**− 0.1508**	−0.1335
LDL	0.0390	0.0963	0.0671	**0.1699**	**0.8891**	**−0.3899**	**1**	**0.3370**	**0.3385**
VLDL	−0.0640	−0.0016	0.0227	0.1252	**0.5973**	**−0.1508**	**0.3370**	**1**	**0.9794**
TG	−0.0558	0.0092	0.0395	**0.1437**	**0.5912**	−0.1335	**0.3385**	**0.9794**	**1**

Values in bold are different from 0 with a significance level alpha = 0.05

**Table 7:** Correlation of adipokines according to BMI category

BMI Category	Variables	ADP	LP	RTN	VF
**Normal Weight**	ADP	**1**	0.1259	0.0157	0.1568
	LP	0.1259	**1**	−0.1357	**0.305**
	RTN	0.0157	−0.1357	**1**	**−0.426**
	VF	0.1568	**0.305**	**−0.426**	**1**
**Overweight**	ADP	**1**	**−0.6234**	**−0.3162**	− 0.2106
	LP	**−0.6234**	**1**	**0.5556**	**0.4342**
	RTN	**−0.3162**	**0.5556**	**1**	**0.3758**
	VF	−0.2106	**0.4342**	**0.3758**	**1**
**Obese**	ADP	**1**	**−0.2559**	−0.0167	0.031
	LP	**−0.2559**	**1**	**0.5853**	**0.497**
	RTN	−0.0167	**0.5853**	**1**	**0.4751**
	VF	0.031	**0.497**	**0.4751**	**1**

Values in bold are different from 0 with a significance level alpha = 0.05

*ADP* Adiponectin, *LP* Leptin, *RTN* Resistin and *VF* Visfatin

BMI classification: Normal Weight = (18.5–24.9 kg/m^2^), Overweight = (25.0–29.99 kg/m^2^), Obese = (Above 30.0 kg/m^2^)

## Discussion

This study was aimed at estimating the level of adiponectin, leptin visfatin, and resistin between 11 and 13 weeks of gestation and to evaluate the effectiveness of using first trimester level of these biomarkers together with maternal characteristics in predicting PE.

This study has shown that leptin was significantly higher in those who subsequently developed PE compared to those without PE. This is in consonance with an earlier study which reported a rise in leptin levels many weeks before the clinical diagnosis of PE [5]. This finding is also similar to a report which indicated an imbalance between adiponectin and leptin in the plasma of women with PE leading to elevated leptin and reduced adiponectin levels; thus these two adipose-derived hormones may be involved in the in the pathogenesis of PE [26]. Similarly leptin has been reported to increase 78% higher at 13 weeks of gestation in women who later developed PE compared to normal pregnant controls [27]. The risk of PE in women whose first trimester leptin levels were ≥ 25 ng/ml were reported to have increased about 18.8 fold compared to pregnant women whose first trimester leptin were < 25 ng/ml [28]. Our results also corroborate other studies which indicated that leptin rises before the clinical onset of the disease [10, 11]. The results of this study and what has been previously reported give an indication of the involvement of leptin in the pathogenesis of PE rather than an increase in leptin as a result of reduced renal clearance. Hyperleptinemia has been reported to increase renal tubular reabsorption of sodium which then leads to water retention resulting in increased blood pressure [29]. Additionally, placental leptin mRNA production is up regulated by tumor necrosis factor (TNF) - α and interleukin (IL)-6 and stimulate the production of endothelin which is a vasoconstrictive peptide [30]. The constriction of the blood vessels leads to high blood pressure leading to PE.

In this study, first trimester adiponectin levels were significantly lower in women with PE compared with the control. This is similar to other studies suggesting that adiponectin levels are inversely related to coronary artery disease but are not strongly related to blood pressure values [31]. In another study, the median maternal high molecular weight and low molecular weight adiponectin concentrations were lower in patients with preeclampsia than in those with normal pregnancies [32]. Previous reports have demonstrated lower first trimester adiponectin levels in women who subsequently developed PE compared to their peers [33, 34]. This study is however contrary to a report indicating that circulating levels of adiponectin was increased in preeclamptic patients compared with normal pregnant women [35, 36]. Another study showed that third trimester adiponectin levels were elevated by almost 50% in women with preeclampsia, as compared to their normotensive peers [37]. Similarly, a cross-sectional study has noted increased circulating levels of adiponectin in women with preeclampsia [36]. These increases which normally occur after first trimester could be as a result of a compensatory feedback mechanisms to the metabolically altered, anti-angiogenic and pro-atherogenic state of severe preeclampsia [36]. The hypoadiponectinemia during first trimester of pregnant women who subsequently developed PE suggests the involvement of this adipocytokine in the pathogenesis of PE. Adiponectin gene expression is inhibited by β-adrenergic stimulation, glucocorticoids and TNF-α [38, 39]. Pregnancy is an inflammatory state associated with increased plasma TNF-α and this could lead to further decline in adiponectin concentration. The increased level of TNF-α results in elevated level of endothelin [30] which constricts the blood vessels leading to high blood pressure [30]. Available data suggests that adiponectin antagonizes the production of angiotensin II [40], therefore with the reduction of adiponectin, there will be overproduction angiotensin II leading to high level of aldosterone. The increased level of aldosterone leads to sodium and water retention resulting in hypertension.

This study revealed significantly higher resistin levels in the pregnancies that resulted in PE compared to those without PE. This is similar to a previous study which indicated increased levels of some selected adipokines including resistin in preeclamptic pregnancies compared to a healthy pregnant group [9]. However, other studies did not show significant difference in resistin levels between women with preeclampsia and healthy pregnant women [41, 42]. Some researchers have reported that women with PE had resistin levels significantly lower than normotensive women of the same gestational age [14]. The rise in resistin levels months before the clinical diagnosis of PE is indicative of the involvement of resistin in the pathophysiology of PE. Resistin concentration in the blood has been found to be associated with coronary artery disease [43]. Available data suggests that resistin concentration in the blood is associated with many inflammatory markers including C-reactive protein, soluble TNF-α receptor-2, IL-6 and lipoprotein-associated phospholipase A2 [43]. The rise in TNF-α receptor-2 and IL-6 concentration lead to increased level of endothelin leading to high blood pressure [30].

Our study has shown that plasma visfatin was increased significantly during PE. The increase in visfatin during PE was evident from the first trimester indicating the possible role of visfatin in the pathogenesis of PE. Visfatin is expressed abundantly in adipose tissue as well as in the placenta and fetal membrane [44]. Median concentrations of visfatin during the second and third trimester of normal pregnancy have been reported to be higher than in the first trimester [45] further supporting the fact that this protein is produced by the placenta and the fetal membrane. Thus, it is possible that this normal production of visfatin is regulated in such a way as to support the growing fetus, however, in some pregnancies; this supportive role of visfatin may be disrupted leading to PE. Our results are similar to a report by some researchers [46] which indicated that higher visfatin levels were found in the PE compared to normal pregnancy. One report indicated no significant differences between normal and pregnancies complicated by preeclampsia [18] while another reported lower levels [17]. The differences in visfatin levels during pregnancy as reported by different researchers could be due to differences in sampling methods, ethnic or geographical differences and possibly the specific assay methods employed. The findings in this study suggest a rise in visfatin concentration before the onset of preeclampsia. The potential of visfatin as a marker of preeclampsia especially in obese women will require further evaluation using larger sample size. Such studies will provide further useful information on prediction of this condition in order to help initiate intervention programs to mitigate the effect PE on maternal and fetal morbidity and mortality.

The fact that after controlling for family history of hypertension (Table 4), the AUCs and the respective sensitivities and specificities did not significantly change indicate that these biomarkers are capable of independently predicting PE irrespective family history of hypertension. Controlling for maternal weight (Table 3), revealed that these adipokines are incapable of predicting PE in women within the normal weight category (BMI between 18.5–24.9 kg/m^2^), however the fact that the overweight category (BMI; 25–29.9 kg/m^2^) performed better in the predictive ability of these adipocytokines than the obese group (BMI ≥30.0 kg/m^2^), indicates possible negative feedback mechanism which tends to reduce plasma concentration of these peptides as weight increases beyond certain threshold. Further studies with larger sample size are required to explain this phenomenon. This study has shown that overweight women are more likely to develop PE during the course of the pregnancy than normal weight pregnant women and corroborates an earlier study which indicated that the risk of developing PE increases about two-to-three fold in women with higher BMI [47] and also similar to another study which associated higher maternal BMI to a number of pregnancy complications including PE [48]. Furthermore, this study also agrees with previous report demonstrating that risk factors for preeclampsia were a maternal age of 35 years and above [49] as well as a BMI greater than 30 kg/m^2^ [50]. The observation in this study suggests the involvement obesity in the pathogenesis of PE. Obesity impairs nitric oxide and endothelial dysfunction [51] and so in a situation where there is excessive accumulation of fat in a pregnant woman it may lead to hypertension and consequently PE could develop.

This study did not show any significant difference in lipids between women who subsequently developed PE compared to their peers with exception of HDL cholesterol which was significantly lower in the PE group (Table 1) compared to the normotensive group. The finding in this study is contrary to a report by researchers in Brazil who reported significant difference in TG rich proteins (VLDL 1) and small dense lipoprotein (LDL III) in women with PE compared to normal pregnant women [52]. Our results also differ from what was reported in the Cape Coast in the central region of Ghana in which the investigators reported significant dyslipidemia in women with PE compared to women without PE [21]. The differences could be due to differences in period of gestation during which sampling was done. Samples for this study were taken before the onset of PE while the samples for the other studies were taken after the onset the disease. The fact that first trimester lipids did not show any significant difference between those who subsequently developed PE and those who did not, suggests that the atherogenic lipid profile generally observed in pregnant women as reported by various researchers may be insufficient in predicting the likelihood of developing PE. It is however, possible that the significantly lower HDL observed among those who subsequently developed PE could be linked to the pathogenesis of the disease since lower HDL is a significant risk factor for hypertension [53].

Taking most of the factors associated with PE and adjusting for these potential confounding variables (age, parity, BMI, family history of diabetes and preeclampsia), adiponectin and resistin were found to be significant and better predictors of PE than leptin and visfatin. Adiponectin decreases the production of angiotensin II [40] and thereby promotes high blood pressure while resistin is associated with increases in TNF-α receptor-2 and IL-6 [43], leading to increased level of endothelin which constricts blood vessels and raises blood pressure [30]. Obesity and parity of four and above were found to be a significant confounders for PE.

### Study limitations

Limitations of the present study were the small sample size and insufficient information about nutritional status of participants. Leafy greens like spinach and klae as well as berries and red beets have high potassium levels. Potassium acts on the kidneys and facilitates excretion of sodium through the kidneys thereby lowering blood pressure. The small sample size may be insufficient in drawing conclusion on the relationship between these adipocytokines and preeclampsia and also the nutritional status and medications could be not be controlled in multivariate analysis.

## Conclusions

Low adiponectin and high leptin, resistin and visfatin were found to be significant predictors of PE with resistin being the best predictor when stratified into the BMI categories. However, adiponectin was the best predictor after controlling for age, parity, BMI, and family history of diabetes and preeclampsia.

## Data Availability

The datasets used and/or analyzed during the current study are available from the corresponding author on reasonable request.

## Abbreviations

BMI: Body mass index
PE: Preeclampsia
ADP: Adiponectin
LP: Leptin
RTN: Resistin
VF: Visfatin
TG: Triglycerides
TC: Total cholesterol
HDL: High density lipoprotein cholesterol
LDL: Low density lipoprotein cholesterol
VLDL: Very low density lipoprotein cholesterol

## References

[1] Kirkendall WM, Burton AC, Epstein FH, Freis ED (1967). Recommendations for human blood pressure determination by sphygmomanometers. Circulation..

[2] Backes CH, Markham K, Moorehead P, Cordero L, Nankervis CA, Giannone PJ. Maternal preeclampsia and neonatal outcomes. J Preg. 2011;4(1):2011–21. 10.1155/2011/214365 PMC3087144 21547086

[3] Wagner LK (2004). Diagnosis and management of preeclampsia. Am Fam Physician.

[4] Obed S, Aniteye P. Pregnancy following eclampsia: a longitudinal study at Korle-BU teaching hospital. Ghana Med J. 2007;41(3):1–4. 10.4314/gmj.v41i3.55282 PMC2279084 18470332

[5] Owiredu W (2008). The prevalence of the metabolic syndrome among Ghanaian pregnancy-induced hypertensive patients using the World Health Organisation and the National Cholesterol Education program III criteria. J Med Sci.

[6] Yeboah FA, Ngala RA, Bawah AT, Asare-Anane H, Alidu H, Hamid A-WM (2017). Adiposity and hyperleptinemia during the first trimester among pregnant women with preeclampsia. Int J Women's Health.

[7] Miehle K, Stepan H, Fasshauer M (2012). Leptin, adiponectin and other adipokines in gestational diabetes mellitus and pre-eclampsia. Clin Endocrinol.

[8] Hendler I, Blackwell SC, Mehta SH, Whitty JE, Russell E, Sorokin Y (2005). The levels of leptin, adiponectin, and resistin in normal weight, overweight, and obese pregnant women with and without preeclampsia. Am J Obstet Gynecol.

[9] Kajantie E, Kaaja R, Ylikorkala O, Andersson S, Laivouri H (2005). Adiponectin concentrations in maternal serum: elevated in preeclampsis but unrelated to insulin sensitivity. J Soc Gynecol Investig.

[10] Haugen F, Ranheim T, Harsem NK, Lips E, Staff AC, Drevon CA (2006). Increased plasma levels of adipokines in preeclampsia: relationship to placenta and adipose tissue gene expression. Am J Physiol Endocrinol Metab.

[11] Anim-Nyame N, Sooranna S, Steer P, Johnson M (2000). Longitudinal analysis of maternal plasma leptin concentrations during normal pregnancy and pre-eclampsia. Hum Reprod.

[12] Chappell LC, Seed PT, Briley A, Kelly FJ, Hunt BJ, Charnock-Jones DS (2002). A longitudinal study of biochemical variables in women at risk of preeclampsia. Am J Obstet Gynecol.

[13] Schubring C, Englaro P, Siebler T, Blum W, Demirakca T, Kratzsch J (1998). Longitudinal analysis of maternal serum leptin levels during pregnancy, at birth and up to six weeks after birth: relation to body mass index, skinfolds, sex steroids and umbilical cord blood leptin levels. Horm Res Paediatrics.

[14] Martinez-Abundis E, Gonzalez-Ortiz M, Pascoe-Gonzalez S (2000). Serum leptin levels and the severity of preeclampsia. Arch Gynecol Obstet.

[15] Cortelazzi D, Corbetta S, Ronzoni S, Pelle F, Marconi A, Cozzi V (2007). Maternal and foetal resistin and adiponectin concentrations in normal and complicated pregnancies. Clin Endocrinol.

[16] Kielstein JT, Becker B, Graf S, Brabant G, Haller H, Fliser D (2003). Increased resistin blood levels are not associated with insulin resistance in patients with renal disease. Am J Kidney Dis.

[17] Fasshauer M, Waldeyer T, Seeger J, Schrey S, Ebert T, Kratzsch J (2008). Serum levels of the adipokine visfatin are increased in pre-eclampsia. Clin Endocrinol.

[18] Hu W, Wang Z, Wang H, Huang H, Dong M (2008). Serum visfatin levels in late pregnancy and pre-eclampsia. Acta Obstet Gynecol Scand.

[19] Mazaki-Tovi S, Vaisbuch E, Romero R, Kusanovic JP, Chaiworapongsa T, Kim SK (2010). Maternal and neonatal circulating visfatin concentrations in patients with pre-eclampsia and a small-for-gestational age neonate. J Matern Fetal Neonatal Med.

[20] Salameh WA, Mastrogiannis DS (1994). Maternal hyperlipidemia in pregnancy. Clin Obstet Gynecol.

[21] Gürsoy A, Kulaksizoglu M, Sahin M, Ertugrul DT, Ozer F, Tutuncu NB (2006). Severe hypertriglyceridemia-induced pancreatitis during pregnancy. J Natl Med Assoc.

[22] Ephraim RK, Doe P, Amoah S, Antoh E (2014). Lipid profile and high maternal body mass index is associated with preeclampsia: a case-control study of the Cape Coast Metropolis. Ann Med Health Sci Res.

[23] Yeboah F, Ngala R, Bawah A, Mbroh H (2016). Maternal adiposity and serum leptin levels at 11-13 weeks of gestation among pregnant women with gestational diabetes mellitus. Int J Med Health Sci.

[24] American College of Obstetricians and Gynecologists (2013). Hypertension in pregnancy. Report of the American College of Obstetricians and Gynecologists’ Task Force on hypertension in pregnancy. Obstet Gynecol.

[25] Jakobsdottir J, Gorin MB, Conley YP, Ferrell RE, Weeks DE (2009). Interpretation of genetic association studies: markers with replicated highly significant odds ratios may be poor classifiers. PLoS Genet.

[26] Ouyang Y, Chen H, Chen H (2007). Reduced plasma adiponectin and elevated leptin in pre-eclampsia. Int J Gynecol Obstet.

[27] Ning Y, Williams M, Muy-Rivera M, Leisenring W, Luthy D (2004). Relationship of maternal plasma leptin and risk of pre-eclampsia: a prospective study. J Matern Fetal Neonatal Med.

[28] Samolis S, Papastefanou I, Panagopoulos P, Galazios G, Kouskoukis A, Maroulis G (2010). Relation between first trimester maternal serum leptin levels and body mass index in normotensive and pre-eclamptic pregnancies–role of leptin as a marker of pre-eclampsia: a prospective case–control study. Gynecol Endocrinol.

[29] Hall JE, Hildebrandt DA, Kuo J (2001). Obesity hypertension: role of leptin and sympathetic nervous system. Am J Hypertens.

[30] Craici IM, Wagner SJ, Weissgerber TL, Grande JP, Garovic VD (2014). Advances in the pathophysiology of pre-eclampsia and related podocyte injury. Kidney Int.

[31] Cesari M, Pessina A, Zanchetta M, De Toni R, Avogaro A, Pedon L (2006). Low plasma adiponectin is associated with coronary artery disease but not with hypertension in high-risk nondiabetic patients. J Intern Med.

[32] Mazaki-Tovi S, Romero R, Vaisbuch E, Erez O, Mittal P, Chaiworapongsa T, Kim SK, Pacora P, Yeo L, Gotsch F (2009). Maternal serum adiponectin multimers in gestational diabetes. J Perinat Med.

[33] D’Anna R, Baviera G, Corrado F, Giordano D, De Vivo A, Nicocia G (2006). Adiponectin and insulin resistance in early-and late-onset pre-eclampsia. BJOG.

[34] D'Anna R, Baviera G, Corrado F, Giordano D, Di Benedetto A, Jasonni VM (2005). Plasma adiponectin concentration in early pregnancy and subsequent risk of hypertensive disorders. Obstet Gynecol.

[35] Khosrowbeygi A, Ahmadvand H. Maternal serum levels of adiponectin in preeclampsia. J Ayub Med Coll Abbottabad. 2009;21(3):79–82. 20929020

[36] Nien JK, Mazaki-Tovi S, Romero R, Erez O, Kusanovic JP, Gotsch F (2007). Adiponectin in severe preeclampsia. J Perinat Med.

[37] Ramsay JE, Jamieson N, Greer IA, Sattar N (2003). Paradoxical elevation in adiponectin concentrations in women with preeclampsia. Hypertension.

[38] Fasshauer M, Klein J, Neumann S, Eszlinger M, Paschke R (2001). Adiponectin gene expression is inhibited by β-adrenergic stimulation via protein kinase a in 3T3-L1 adipocytes. FEBS Lett.

[39] Fasshauer M, Klein J, Neumann S, Eszlinger M, Paschke R (2002). Hormonal regulation of adiponectin gene expression in 3T3-L1 adipocytes. Biochem Biophys Res Commun.

[40] Kintscher U, Unger T (2005). Vascular protection in diabetes: a pharmacological view of angiotensin II type 1 receptor blockers. Acta Diabetol.

[41] Danqing C, Minyue D, Qin F, Jing H, Zhengping W, Xiaofu Y (2005). Alterations of serum resistin in normal pregnancy and pre-eclampsia. Clin Sci.

[42] Chen D, Shi Z, Dong M, Fang Q, He J, Wang Z (2005). Relationship between serum resistin level and preeclampsia. Zhejiang Da Xue Bao Yi Xue Ban.

[43] Reilly MP, Lehrke M, Wolfe ML, Rohatgi A, Lazar MA, Rader DJ (2005). Resistin is an inflammatory marker of atherosclerosis in humans. Circulation.

[44] Kendal-Wright C, Hubbard D, Bryant-Greenwood G (2008). Chronic stretching of amniotic epithelial cells increases pre-B cell colony-enhancing factor (PBEF/visfatin) expression and protects them from apoptosis. Placenta.

[45] Mastorakos G, Valsamakis G, Papatheodorou DC, Barlas I, Margeli A, Boutsiadis A (2007). The role of adipocytokines in insulin resistance in normal pregnancy: visfatin concentrations in early pregnancy predict insulin sensitivity. Clin Chem.

[46] Fasshauer M, Blüher M, Stumvoll M, Tönessen P, Faber R, Stepan H (2007). Differential regulation of visfatin and adiponectin in pregnancies with normal and abnormal placental function. Clin Endocrinol.

[47] Bodnar LM, Ness RB, Markovic N, Roberts JM (2005). The risk of preeclampsia rises with increasing prepregnancy body mass index. Ann Epidemiol.

[48] Cedergren MI (2004). Maternal morbid obesity and the risk of adverse pregnancy outcome. Obstet Gynecol.

[49] Conde-Agudelo A, Belizán JM (2000). Risk factors for pre-eclampsia in a large cohort of Latin American and Caribbean women. BJOG.

[50] Luealon P, Phupong V (2010). Risk factors of preeclampsia in Thai women. J Med Assoc Thail.

[51] Frühbeck G (1999). Pivotal role of nitric oxide in the control of blood pressure after leptin administration. Diabetes.

[52] Lima VJ, Andrade CR, Ruschi GE, Sass N (2011). Serum lipid levels in pregnancies complicated by preeclampsia. Sao Paulo Med J.

[53] Onat A, Hergenç G, Sarı I, Türkmen S, Can G, Sansoy V (2005). Dyslipidemic hypertension: distinctive features and cardiovascular risk in a prospective population-based study. Am J Hypertens.

